# Calorie restriction effects on circadian rhythms in gene expression are sex dependent

**DOI:** 10.1038/s41598-017-09289-9

**Published:** 2017-08-29

**Authors:** Artem A. Astafev, Sonal A. Patel, Roman V. Kondratov

**Affiliations:** Department of Biological, Geological, and Environmental Sciences and Center for Gene Regulation in Health and Diseases, Cleveland State University, Cleveland, OH 44115 USA

## Abstract

The rhythms in the expression of circadian clock genes are affected by calorie restriction (CR), a dietary paradigm known to increase lifespan. Many physiological effects of CR differ between males and females; here we investigated if the sex of animals affects the CR induced changes in the circadian rhythms. The liver expression of some circadian clock genes such as *Bmal1* and three *Periods* (*Per1*, *Per2* and *Per3*) and the effect of CR on the expression of these genes were sex independent, while the expression of *Rev-Erb alpha*, *Ror gamma* and both *Cryptochome* (*Cry1* and *Cry2*) genes was different between males and females. The effect of CR on *Rev-Erb alpha*, *Ror gamma* and *Cry1* gene expression was sex dependent. The expression and the effects of CR were sex-specific for several genes previously reported to be regulated by CR: *Fmo3*, *Mup4*, *Serpina12* and *Cyp4a12*, while the expression of *Cyp4a14a* was sex independent. IGF signaling plays an important role in aging and CR effects. *Igf-1* expression is regulated by CR and by the circadian clock, we found that rhythms in *Igf-1* expression have sexual dimorphism. Our data provide molecular evidence that the sex of animals is an important modulator of circadian rhythms in gene expression and their response to CR.

## Introduction

The increase in lifespan and delay of aging by caloric restriction (CR) is well documented in many organisms, including mammals^1^. CR affects many physiological systems - it causes changes in the levels of many hormones, improves glucose homeostasis, mitochondrial functioning and proteostasis, increases resistance to stress and reduces incidence of cancer^2^. All these changes are considered beneficial for health and longevity. Several signaling pathways, most of which are well conserved, such as TOR, sirtuin and insulin/IGF signaling pathways, are implicated in the mechanisms of CR^3, 4^. Targeting some of these pathways pharmacologically might mimic the effects of CR and lead to increased lifespan^5, 6^. Recently, us and other researchers reported that CR affects the daily rhythms in the expression of several circadian clock genes in mammals and flies and proposed that circadian clocks might be involved in CR mechanisms^7, 8^.

The circadian system or circadian clock generates 24 hour rhythms in physiology and metabolism in a variety of organisms across taxa^9, 10^. The clock is important for the synchronization of different processes inside the organism with a periodic 24-hour light/dark environmental cycle. Epidemiological data on the risk of development of many diseases such as diabetes, cardiovascular diseases and cancer in shift workers^11, 12^, as well as the development of similar pathologies in animal models of clock disruption support the importance of circadian clocks and rhythms in health^9, 13, 14^. In mammals, the circadian clocks operate in most of the tissues and organs. The central clock in the suprachiasmatic nucleus (SCN) of the hypothalamus is entrained by the periodic light/dark cycle and it regulates the clocks in other organs (called peripheral clocks)^9, 11, 15^. The peripheral clocks generate rhythms in gene expression and metabolism. On a cellular level circadian rhythms are generated through the activity of interacting feedback loops formed by a dozen of core clock genes and their products. Transcriptional factors CLOCK and BMAL1 drive the expression of *Period* (*Pers*) and *Cryptochrome* (*Crys*) genes; PERs and CRYs proteins inhibit CLOCK:BMAL1 transcriptional activity and, as a result, their own expression, thus forming the negative feedback loop. The products of another group of CLOCK:BMAL1 regulated genes, *Rev-Erbs* and *Rors*, in turn negatively or positively control *Bmal1* transcription, therefore forming another negative and positive feedback loop^9, 11, 13, 16^. In addition to regulation by SCN derived signals, the peripheral clocks can be reset by some other cues, with food being the most efficient regulator^10, 17^. CR is a dietary paradigm in which the amount of the food is reduced. For mammals the food is typically provided in a periodic circadian manner. We reported previously that 30% CR affects the amplitude and absolute level of expression for several circadian clock genes^7^. The effect was observed on both mRNA and protein levels. Importantly, CR fails to provide all physiological benefits including the effect on longevity in mice deficient for the circadian transcriptional factor BMAL1^3^. Similarly, CR does not have full effects on the increase in lifespan in Drosophila flies with genetic ablation of some clock genes^8^. Thus, circadian clocks might represent a conserved physiological system essential for CR mechanisms, which warrants a further study on interaction between circadian clocks and CR.

In the previous studies the effect of CR on clock gene expression was investigated only in male mice. The majority of the studies on circadian clocks and rhythms either did not compare males to females or did not include females into the experimental setup at all - due to a general bias in biomedical research^18^. Recent data suggest that many effects of CR are sex - dependent^2^ and some circadian rhythms in behavior and physiology are different in males and females^2, 19, 20^. Therefore, in the present report we compared the effect of CR on circadian rhythms in gene expression between male and female mice.

## Results

### Effect of sex on the circadian rhythms in expression of clock genes

**Figure 1:**
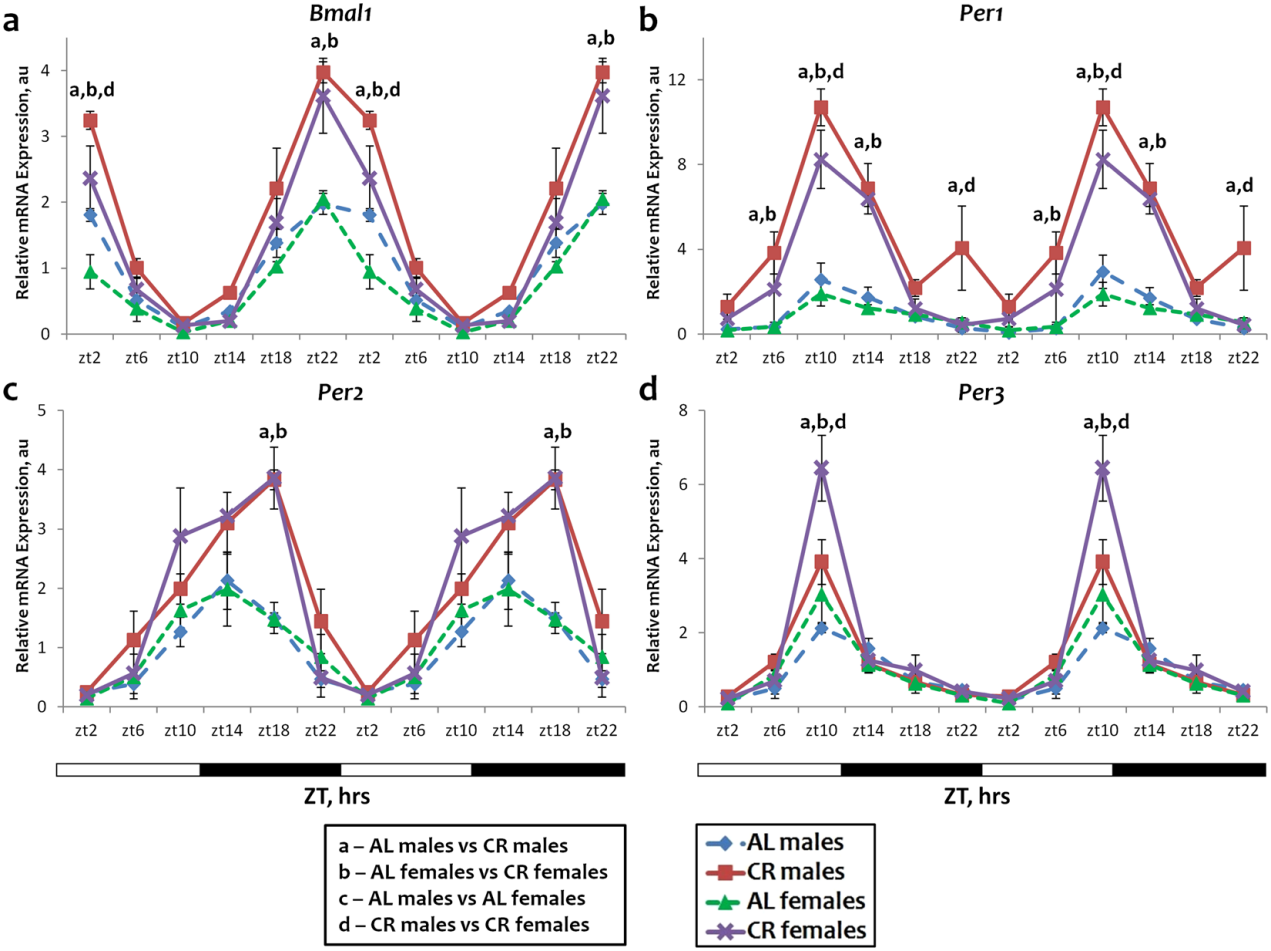
Sex independent expression and response to CR for some circadian clock genes. The daily rhythms in expression of mRNA for *Bmal1* (**a**), *Per1* (**b**), *Per2* (**c**) and *Per3* (**d**) in the liver: blue diamonds and dashed lines – AL male mice; red squares and solid lines – CR male mice, green triangles and dashed lines – AL female mice; purple x and solid lines – CR female mice. For all panels graphs are double plotted. Light is on at ZT0 and off at ZT12. a, b, c, d – statistically significant difference (p < 0.05) between indicated groups at particular time point. Open bars represent light and black bars represent dark phase of the day. Food for CR group was provided at ZT14. At every time of the day 3 mice of each sex were used for each diet.

In the previous study we found that the daily rhythms in the expression of *Bmal1*, *Per1*, *Per2*, *Per3*, *Cry2* and *Ror γ* were significantly affected by CR in the liver of male mice^8^. Male and female mice of the same age (starting at 3 months) were subjected to 30% CR for two subsequent months. The expression of circadian clock genes was assayed in the liver of CR and control animals, which had ad libitum (AL) access to food throughout the day. We found that in the liver of AL mice the expression of *Bmal1* and *Per1*, *Per2 and Per3* genes was similar between males and females, and there were no significant differences in the phase of the expression (see Fig. 1 and Supplementary Table S2). CR significantly induced the expression of these genes: at ZT22 and ZT2 for *Bmal1*; at ZT10 and ZT14 for *Per1*; at ZT18 for *Per2* and ZT10 for *Per3* in both males and females (Fig. 1). While at some time points (ZT2 for *Bmal1*, *ZT10* for *Per1* and *Per3*) we observed a statistically significant difference between the expressions in males and females on CR, the changes in the expression were still observed for both males and females and these changes were towards the same direction. Therefore, we concluded that the sex of animals might have some effects on the magnitude of the response but the expression of *Bmal1*, *Per1*, *Per2* and *Per3* to CR was similar in both sexes. CR did not affect the phase of gene expression because the food for the CR group was provided at ZT14 (2 hours after the light was off), which is the physiological feeding time for nocturnal rodents.

**Figure 2:**
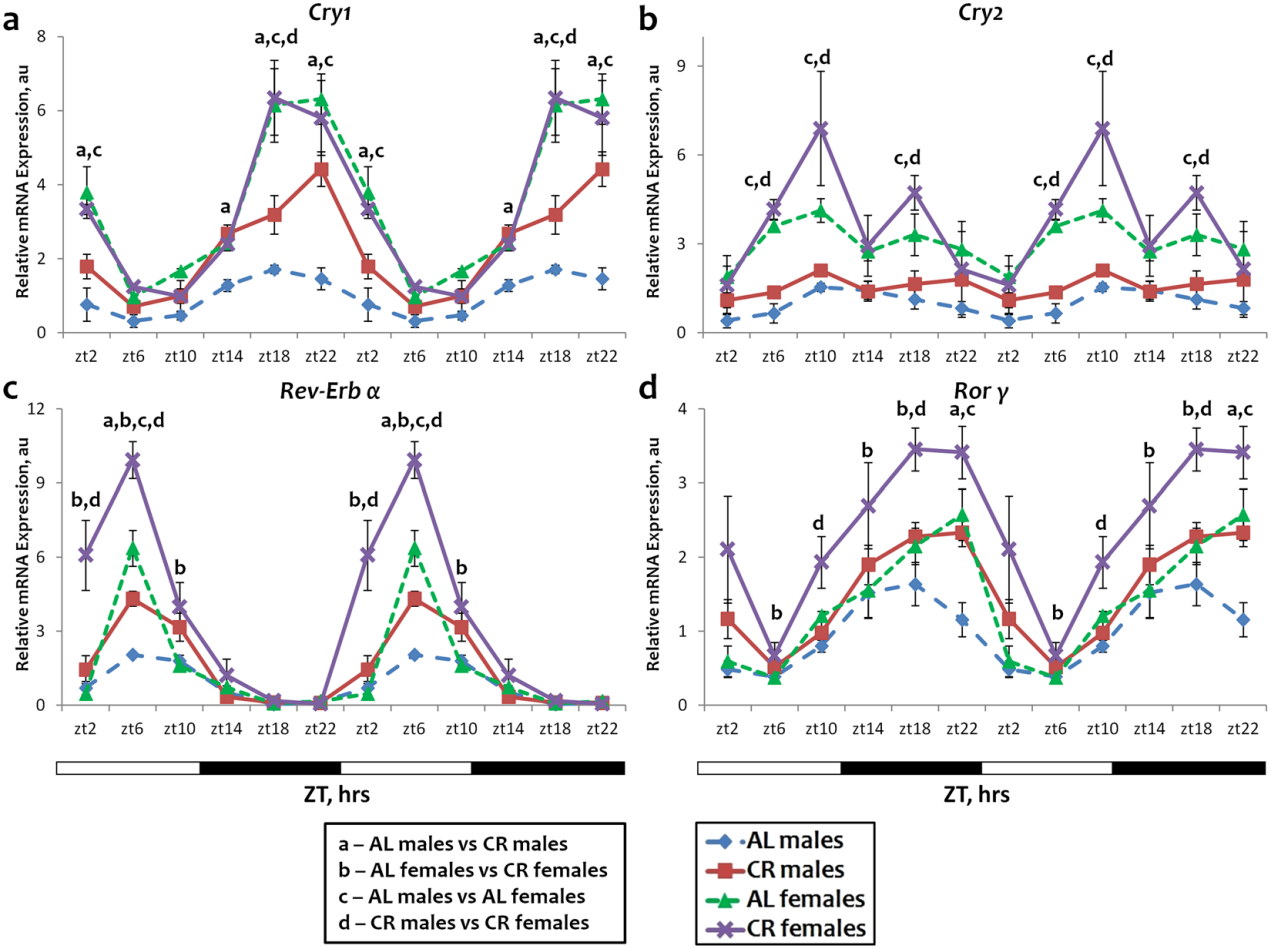
Sexual dimorphism in the expression and response to CR for some circadian clock genes. The daily rhythms in expression of mRNA for *Cry1* (**a**), *Cry2* (**b**), *Rev-Erb α* (**c**) and *Ror γ* (**d**) in the liver: blue diamonds and dashed lines – AL male mice; red squares and solid lines – CR male mice, green triangles and dashed lines – AL female mice; purple x and solid lines – CR female mice. For all panels graphs are double plotted. Light is on at ZT0 and off at ZT12. a, b, c, d – statistically significant difference (p < 0.05) between indicated groups at time point. Open bars represent light and black bars represent dark phase of the day. Food for CR group was provided at ZT14. At every time of the day 3 mice of each sex were used for each diet.

The expression of *Cry1*, *Cry2* and *Rev-Erb α* and *Ror γ* was significantly affected by the sex of the mice. In the AL group the expression of all four genes was significantly higher in the liver of female mice: at ZT18, ZT22 and ZT2 for Cry1 (Fig. 2a); at ZT6, ZT10 and ZT18 for *Cry2* (Fig. 2b); at ZT6 for *Rev-Erb α* (Fig. 2c); at ZT22 for *Ror γ* (Fig. 2d). The effect of CR on the expression of these genes was also affected by the sex. The expression of the *Cry1* gene was induced by CR at several time points (ZT14-ZT2) in males but CR did not have any effect on the expression of *Cry1* in females (Fig. 2a). By contrast, the expression of *Cry2* was not significantly affected by CR neither in males, in agreement with our previous work, nor in females (Fig. 2b). It is worth noting, according to the rhythmic analysis, that the expression of *Cry2* in males was rhythmic under AL and arrhythmic upon CR (Supplementary Table S2). *Rev-Erb α* expression was induced by CR in males only at one time point (ZT6), while in females significant induction was detected at three time points ZT2, ZT6, ZT10 (Fig. 2c). A similar pattern of response to CR was observed in the expression of *Ror γγ* gene (Fig. 2d). *Ror γ* expression was induced by CR in males, again, at only one time point (ZT22) while in females significant induction was detected at three time points: ZT6, ZT14, ZT18 (Fig. 2d). Thus, out of four clock genes, expressions of which were showing sexual dimorphism in the liver, CR affected the expression of one (*Cry1*) only in males; CR affected the expression of two (*Rev-Erb α*, *Ror γ*) in both sexes, but in females the induction was stronger and observed at several time points; finally, CR did not affect the expression of one of these genes (*Cry2*) in either sex.

### Effect of sex on the circadian rhythms in the expression of CR regulated genes

**Figure 3:**
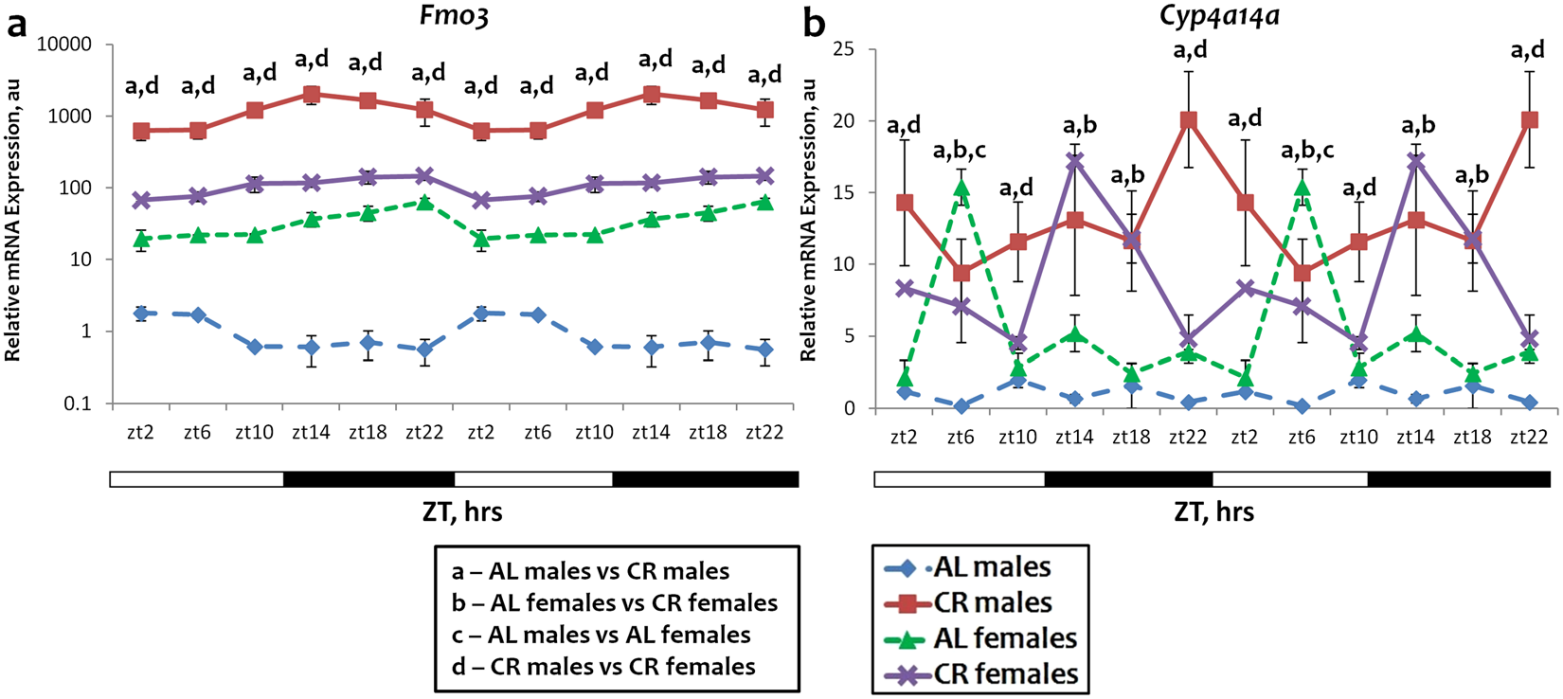
Sexual dimorphism and sex independent expression and response to CR for genes upregulated by CR. The daily rhythms in expression of mRNA for *Fmo3* (**a**) and *Cyp4a14a* (**b**) in the liver: blue diamonds and dashed lines – AL male mice; red squares and solid lines – CR male mice, green triangles and dashed lines – AL female mice; purple x and solid lines – CR female mice. For all panels graphs are double plotted. Light is on at ZT0 and off at ZT12. a, b, c, d – statistically significant difference (p < 0.05) between indicated groups at time point. Open bars represent light and black bars represent dark phase of the day. Food for CR group was provided at ZT14. At every time of the day 3 mice of each sex were used for each diet.

**Figure 4:**
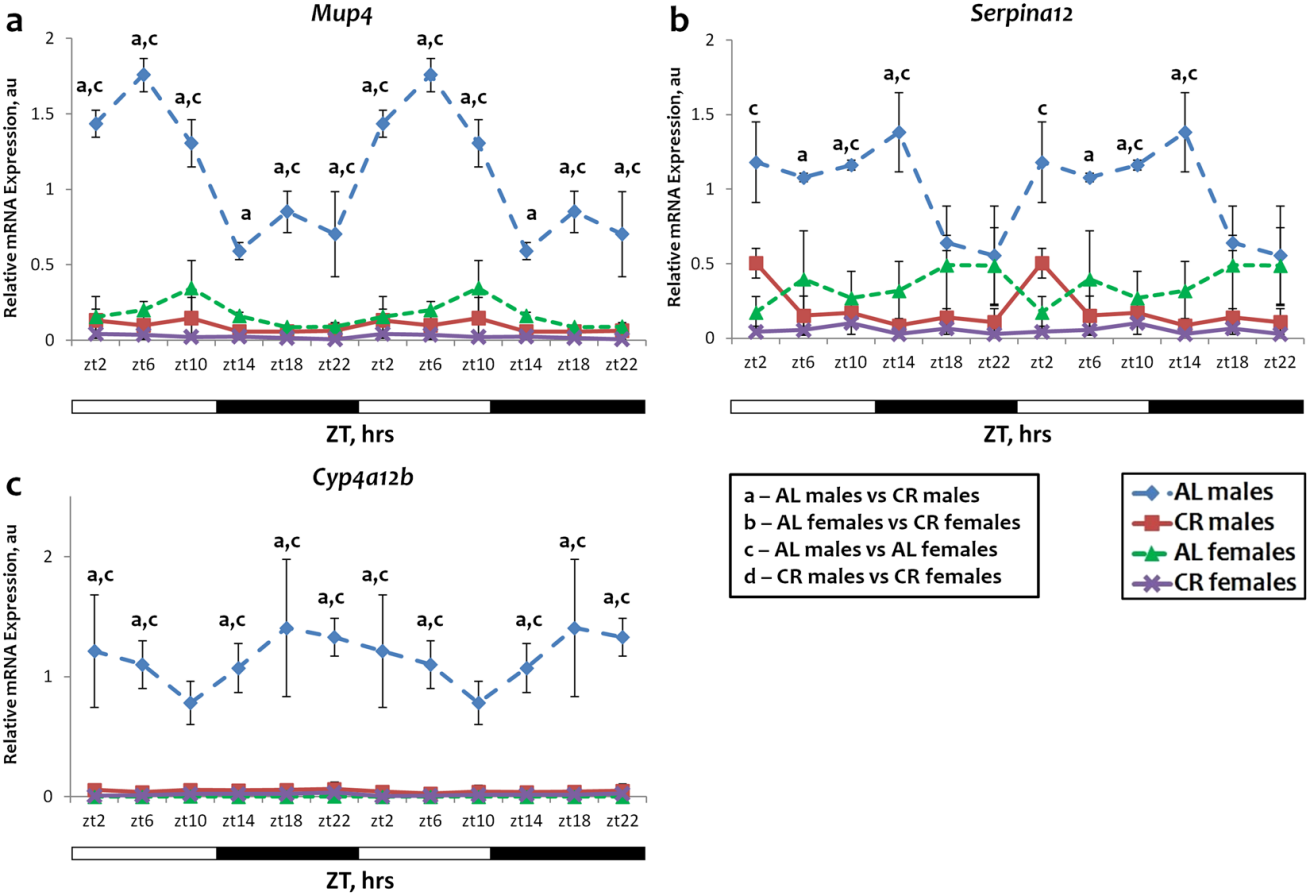
Sexual dimorphism and sex independent expression and response to CR for genes downregulated by CR. The daily rhythms in expression of mRNA for *Mup4* (**a**), *Serpina12* (**b**) and *Cyp4a12b* (**c**) and in the liver: blue diamonds and dashed lines – AL male mice; red squares and solid lines – CR male mice, green triangles and dashed lines – AL female mice; purple x and solid lines – CR female mice. For all panels graphs are double plotted. Light is on at ZT0 and off at ZT12. a, b, c, d – statistically significant difference (p < 0.05) between indicated groups at time point. Open bars represent light and black bars represent dark phase of the day. Food for CR group was provided at ZT14. At every time of the day 3 mice of each sex were used for each diet.

Several genes have been identified through previous studies as targets of CR and proposed as longevity candidate genes^21^. We have found that in the liver their expression is regulated exclusively by CR but not by time restricted feeding or fasting^7^. It was also reported that the expression of some of them is sexually dimorphic. We analyzed the expression of *Fmo3*, *Serpina12*, *Mup4*, *Cyp4a12b* and *Cyp4a14a*. The results are presented in Figs 3 and 4. The expressions of all these genes showed sexual dimorphism: *Fmo3*, *Mup4* and *Cyp4a12b* expression was different between sexes at all time points throughout the day; *Serpina12* expression was different between males and females at ZT2, ZT10-14 (Fig. 4b) and *Cyp4a14a* expression differed only at ZT6 (Fig. 3b). Under AL conditions *Fmo3* expression was 20–100 fold (depending on the time of the day) higher in females than in males (Fig. 3a). These results are in agreement with the previously reported sexual dimorphism of *Fmo3* expression^22, 23^. *Fmo3* expression was induced by CR in both males and females, again in agreement with the previously published data^24^. There was a dramatic difference between sexes in the magnitude of the CR effects on the expression. In females, *Fmo3* expression was induced 2–5 fold, and in males the expression was induced by 600–3000 fold. Due to this difference in the response to CR, under CR conditions *Fmo3* expression was significantly higher in males, while under AL control conditions the expression was higher in females.

The expression of *Serpina12*, *Mup4* and *Cyp4a12b* under AL conditions was several fold higher in males and it was significantly reduced upon CR in males only. The tendency toward reduced expression was observed in females, but the basal level was low and CR did not have a statistically significant effect (Fig. 4). There was no difference in the expression of *Cyp4a14a* gene between males and females at 5 time points under AL conditions, and the expression was higher in females only at ZT6 (Fig. 3b). CR induced the expression in males at all time points throughout the day, however in females the induction was observed only at ZT14 time point and CR rather caused a phase shift in expression pattern of Cyp4a14a in females.

Thus, out of 5 tested genes four: *Fmo3*, *Serpina12*, *Mup4* and *Cyp4a12b* genes have strong sexual dimorphism in expression and for *Cyp4a14a* gene the dimorphism has only been observed at a single time point.

### Effect of sex on the circadian rhythms in protein expression

**Figure 5:**
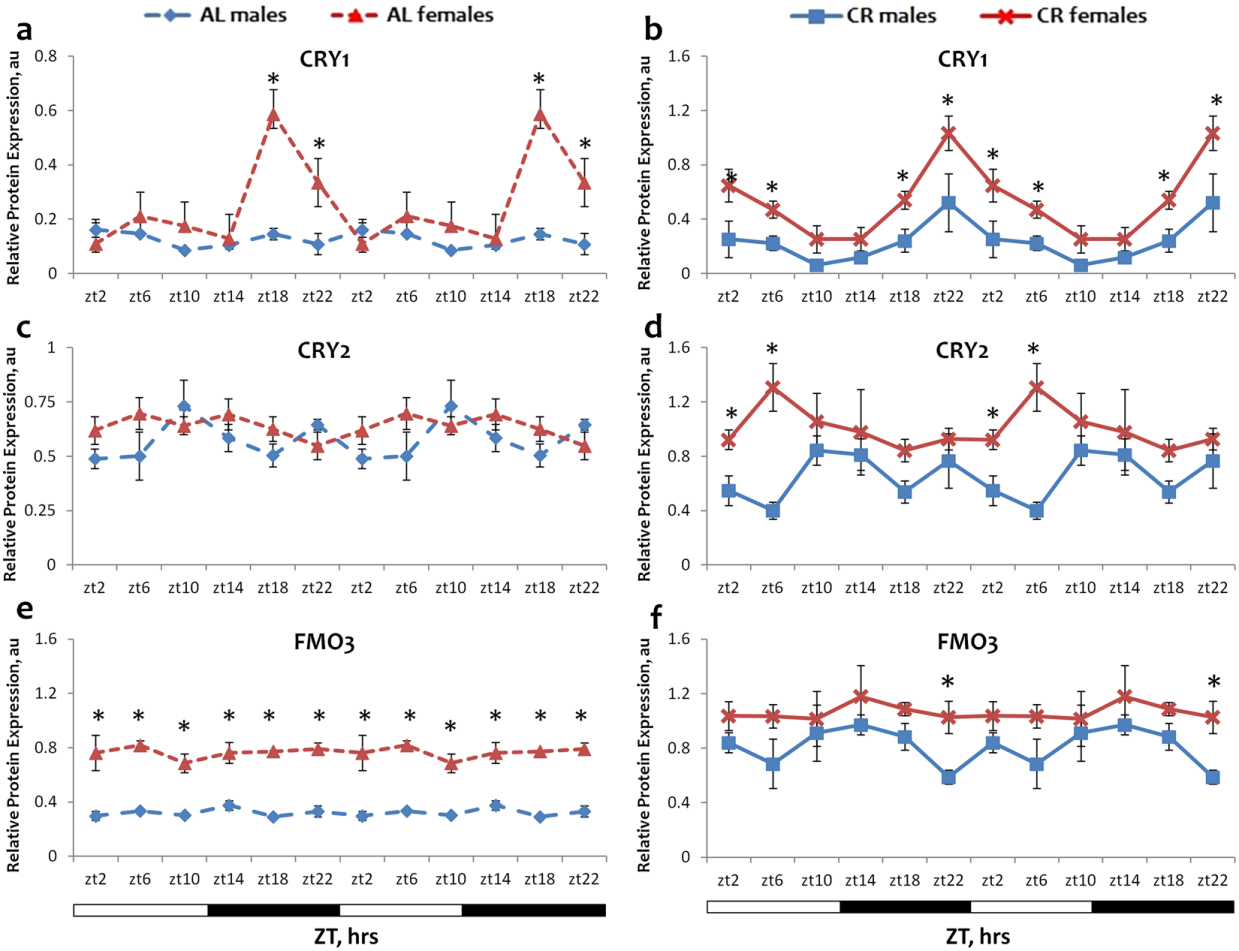
Sexual dimorphism in the expression for CRY1, CRY2 and FMO3 proteins. The daily rhythms in expression of proteins for CRY1 (**a** and **b**), CRY2 (**c** and **d**), FMO3 (**e** and **f**) in the liver: blue diamonds and dashed lines – AL male mice; blue squares and solid lines – CR male mice, red triangles and dashed lines – AL female mice; red x and solid lines – CR female mice. For all panels graphs are double plotted. Light is on at ZT0 and off at ZT12. Asterisk (*) indicates statistically significant difference (p < 0.05) in protein levels between the sexes. Open bars represent light and black bars represent dark phase of the day. Food for CR group was provided at ZT14. At every time of the day 3 mice of each sex were used for each diet. The respective images from Western blot analysis are shown in Supplementary Figs S1 and S2.

To assay whether the observed sexual dimorphism in mRNA expression translates into dimorphism in protein expression, we analyzed the expression of several genes by western blotting. The results are presented on Fig. 5. In agreement with mRNA data the expression of CRY1 protein was significantly higher in female livers under AL and CR conditions. Interestingly, under both AL and CR conditions the sex-based difference in protein levels of CRY1 was similar to the difference observed between sexes on mRNA level – females showed higher protein expression at ZT18-22 under AL and at ZT2-6; ZT18-22 under CR (Fig. 5a,b). The expression of CRY2 was higher in female mice under CR at two time points (Fig. 5d). Under AL conditions the expression of FMO3 protein was several fold higher in female livers than in male at all 6 time points throughout the day (Fig. 5e), again in agreement with the mRNA data. The difference between sexes was not significant for CR samples except for ZT22. (Fig. 5f) Thus, we observed some correlation between mRNA and protein data on sexual dimorphism in the expression of circadian clock and longevity candidate genes. At the same time, an absolute correlation between mRNA and protein data is relatively rare, the protein expression is regulated on multiple levels: translational and posttranslational. The circadian clock is involved in the posttranscriptional control of gene expression on multiple levels.

### Effect of sex on circadian rhythms in *Igf-1* expression

**Figure 6:**
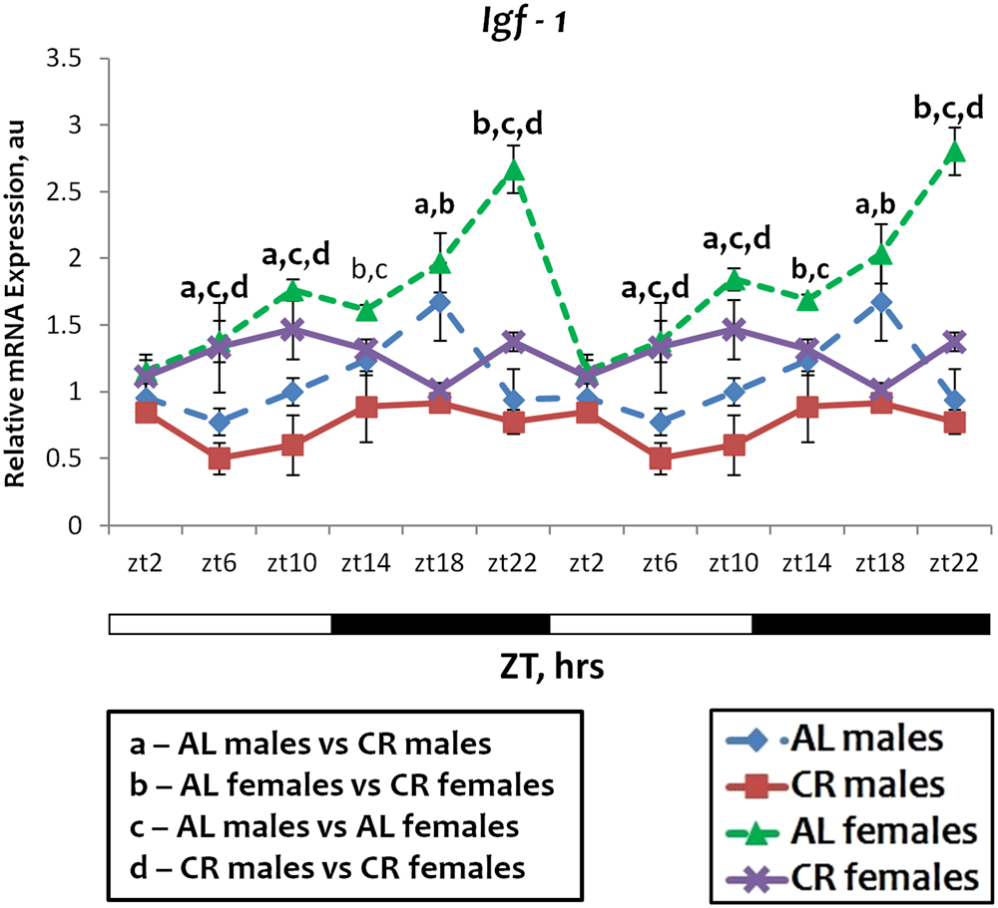
Sexual dimorphism in *Igf-1* mRNA expression. The daily rhythms in expression of mRNA for *Igf-1* in the liver: blue diamonds and dashed lines – AL male mice; red squares and solid lines – CR male mice, green triangles and dashed lines – AL female mice; purple x and solid lines – CR female mice. For all panels graphs are double plotted. Light is on at ZT0 and off at ZT12. a, b, c, d – statistically significant difference (p < 0.05) between indicated groups. Open bars represent light and black bars represent dark phase of the day. Food for CR group was provided at ZT14. At every time of the day 3 mice of each sex were used for each diet.

To extend our observations on sexual dimorphism in circadian rhythms and responses to CR we decided to investigate a signaling pathway relevant to CR, circadian clock and aging. We recently reported that Cryptochromes are involved in the regulation of Insulin like growth factor 1 (*Igf-1*) expression. We assayed the liver expression of *Igf-1* gene in male and female mice on both diets. The results are presented in Fig. 6. We found that *Igf-1* mRNA expression is higher in females than in males under AL conditions. For both sexes, CR caused the reduction in the expression, but the expression in females still remained higher (Fig. 6). Thus, the expression of *Igf-1* demonstrated sexual dimorphism at both diets.

## Discussion

The role of circadian clocks and rhythms in physiology and metabolism are well recognized. The connection between the peripheral circadian clocks and aging is also established. Clock disruption through genetic ablation of clock genes: *Bmal1* and *Clock* results in reduced lifespan in mice^14, 25, 26^ and *Period 2* ablation yields mixed outcomes: under regular conditions mutant mice live longer than wild type^27^ but after whole-body irradiation their lifespan is shorter than that of wild type mice^14^. Clock disruption leads to reduced lifespan in the Drosophila model^8^, which suggests the conservation of clock mechanisms in aging. Recent data also support the hypothesis that circadian clocks are a part of CR-modulated mechanisms. In both Drosophila^8^ and mouse^7^ models, CR significantly affects the rhythms in clock gene expression in peripheral organs, causing the change in the amplitude of the oscillations and the absolute levels of expression. Lifespan extension induced by CR is significantly compromised in flies with clock disruption^8^. In mammals the effect is even more dramatic, CR fails to increase the lifespan of mice deficient for *Bmal1* ^25^. These data together suggest a close interaction between the circadian clocks, CR and aging.

There is more and more evidence that sex strongly influences many physiological processes. Circadian rhythms in physiology and behavior are influenced by sex^2, 18, 20^. Indeed, there is a sex difference in SCN morphology and signaling to and from SCN^19^, which might contribute to the sex difference in many of the central clock controlled processes such as the timing of activity^18^. Sex may also affect the peripheral clocks and the entrainment of SCN independent circadian rhythms such as food anticipatory activity^18, 19^.

Many beneficial effects of CR, including the lifespan increase, are significantly affected by the sex. For example, for C57B/6 J mice (the same strain that was used in our study), 20% CR increases lifespan in females more significantly than in males, while 40% CR increases lifespan in males but not in females^2^. Effects of CR on changes in body temperature, changes in glucose and insulin levels, changes in liver metabolism and gene expression were strongly influenced by the sex of the mice^2^.

Here we compared the effect of CR on the expression of circadian clock genes in the liver between male and female mice on AL and 30% CR diets. We did not find any sexual dimorphism in the expression or response to CR for circadian clock genes *Bmal1*, *Per1*, *Per2* and *Per3*. The expression of several clock genes: *Cry1*, *Cry2*, *Rev-Erb α and Ror γγ* was significantly different between males and females on both diets used. In addition, the effect of CR on the expression of *Cry1*, *Rev-Erb α* and *Ror γ* was sex-dependent.

There are only a few reports on the effects of sex on clock gene expression. A different phase in the expression of *Per2* gene between males and females has been reported for pituitary^28^. Sexual dimorphism in the response to experimental chronic jet lag was observed for several clock genes: *Clock*, *Cry2*, *Rev-Erb α* and *Bmal1*; no sexual dimorphism was observed for *Per1* or *Per2* genes^20^. To the best of our knowledge, there have been no studies investigating the effect of sex on *Cry1* or *Ror γ* expression in circadian manner. What could be the physiological significance of this difference in clock gene expression and response to CR? The sexual dimorphism in liver gene expression depends on transcriptional factor STAT5b. Recently CRYs have been implicated in the regulation of STAT5b activity^29^ and it was also previously reported that CRYs are essential for sexual dimorphism in liver gene expression^30^. Thus, the sexual dimorphism here observed in *Cry* gene expression might be a contributing factor, which warrants further study on the interaction between diet, CRYs and sexual dimorphism.

Sex also affected circadian rhythms in the expression of several genes regulated by CR. Flavin-containing monooxygenase 3 (FMO3) is responsible for the oxidation of trimethylamine (TMA) to trimethylamine-N-oxide (TMAO)^31^. *Fmo3* is known to be one of the genes most strongly induced by CR in liver, and it was reported that *Fmo3* homolog is involved in the determination of lifespan in nematodes^32^. It is also known that *Fmo3* expression is strongly affected by sex in mice, the expression is high in females and practically undetectable in males^33^. In accordance with previously published data, we found that *Fmo3* expression was higher in females than in males under AL conditions and that CR induced the expression in both sexes but with a different magnitude. *Serpina12* gene encodes secreted a serine protease inhibitor from the serpin superfamily. The product is known as vaspin and is implicated in the control of metabolism, insulin sensitivity and glucose homeostasis^34^. The level of vaspin in human blood serum was found to be oscillating in circadian manner^35^ and sexual dimorphism in circulating vaspin levels in obese individuals was also reported^36^. To the best of our knowledge sexual dimorphism in mRNA expression and the dimorphism in response to CR for *Serpina12* gene have not been previously reported. *Mup4* gene encodes major urinary protein 4, which belongs to the family of male-specific proteins that can bind odorant molecules and regulate their release from the scent marks^37^. The observed sexual dimorphism in the expression of *Mup4* was expected and male-specific response to CR was expected as well^38^. *Cyp4a12b* and *Cyp4a14a* genes encode the enzymes that belong to the cytochromeP450 superfamily. These enzymes are involved in omega-hydroxylation of fatty acids and are implicated in the regulation of blood pressure^39, 40^. *Cyp4a12b* demonstrated a strong male-specific expression (Fig. 4) throughout the day, which is in agreement with reports by Jeffery *et al*.^41^ and Zhang *et al*.^42^ It was not reported previously that the effect of CR on the expression of members of the *Cyp4a* family is sex dependent. Igf-1 is an evolutionary conserved regulator of longevity. It is well documented that the Igf-1 expression is reduced in response to CR and might be different between males and females. The sexual dimorphism in the expression of *Igf-1* mRNA for both diets reported here might contribute to the difference in longevity and the effects of CR between sexes.

The expressions of some genes showing sexual dimorphism (*Fmo3*, *Mup4* and *Cyp4a12*) are regulated by gonadal hormones. It is unknown if the expression of circadian clock genes might be regulated by gonadal hormones. Can the observed sexual dimorphism be a simple consequence of the difference in gonadal hormone levels? Several facts contest this simplistic explanation. In agreement with the model of the “feminizing” effect of CR on liver gene *Cry1* expression was induced by CR only in males and *Fmo3* was induced much stronger in males than in females. However, under CR diet the expression of *Fmo3* in the liver of males is tenfold higher than in females; the expression of *Cry2* was affected by CR only in females and the expression of *Rev-Erb α* and *Ror γ* was induced in both sexes. Plus, the CR induced reduction in testosterone levels in males was only about 11%^43^ so testosterone levels were still significantly higher in males than in females. These data suggest the existence of a more complex mechanism of regulation, not just a simple response to changes in gonadal hormone levels.

In summary, there is more and more evidence that sex is an important variable in many physiology functions not necessarily related exclusively to reproduction. Our data on the sexual dimorphism in clock gene expression and sexual dimorphism in CR-induced effects support this opinion. There is some controversy on the benefits of CR, multiple factors might influence the response of the organism to CR and present data suggest that the interaction between sex and circadian rhythms must be taken into consideration for optimal CR implementation.

## Materials and Methods

### Ethics Statement

All the animal studies were performed with approval from the Institutional Animal Care and Use Committee (IACUC) of Cleveland State University (Protocol No. 21124-KON-S). The care and use of mice were carried out in accordance with the guidelines of the Institutional Animal Care and Use Committee (IACUC) of the Cleveland State University.

### Experimental animals

Wild type mice were of C57B6J background. Mice were maintained on the 12:12 light:dark cycle with lights on at 7:00 am and off at 7:00 pm and fed the 18% protein rodent diet (Harlan). The *ad libitum* (AL) group had unrestricted access to food. Calorie restriction (CR) was started at 3 months of age. For the first week of the diet the animals were on 10% restriction, for the second week – on 20% restriction and on 30% restriction for the rest of their life and subsequent experimental measurements. The CR groups received their food once per day at ZT14 (two hours after light off). After two months of CR the tissues were collected for analysis. For AL groups the tissues were collected for 5-month-old (age match) animals. For all groups the tissues were collected at six different time points throughout the day. All groups had unrestricted access to water.

### RNA isolation and analysis of mRNA expression

For gene expression studies, tissues were collected every four hours throughout the day and stored at −80 °C. Total RNA was isolated from the liver using TriZol reagent (Invitrogen, Carlsbad, CA) according to the manufacturer’s protocol. Briefly, frozen liver piece was minced in 1 ml TriZol reagent with pestle on ice. Following chloroform extraction step, total RNA was precipitated with isopropanol by centrifugation. The RNA pellet was dissolved in water and quantified on Nanodrop, RNA quality was checked on 1% agarose gel. The RT mix was prepared using 1 µg of mRNA, 50 ng of random hexamer (N808127, Invitrogen), 10 mM dNTP (DD0058, Biobasic), 0.1 M DDT and RNaseOUT Recombinant RNase inhibitor (10777-019, 40 units/ µl) and it was reverse transcribed using SuperScript III Reverse Transcriptase (18080-044, Invitrogen). RNA quantification was performed using qPCR with Universal SYBR Green Mix (1725125, BioRad). The reaction was carried out in triplicates for the gene of interest and in duplicates for the normalizing control using CFX96 Real-Time PCR Detection System (BioRad) with 50ng of cDNA. Thermal cycling conditions used were according to the instruction of SYBR Green mix protocol and relative mRNA abundance was calculated using the comparative delta-Ct method with ribosomal 18 S rRNA as reference gene as described in^44^. Water was used as negative control for qPCR analysis. Product specificity was confirmed by melting curve analysis while primer pair efficiency was calculated by generating standard curve using serial dilutions of standard. Primers used for the analysis of expression are listed in the Supplementary Table S1.

### Immunoblot analysis

For analysis of protein expression tissues from three mice per every time point were used for both sexes and each feeding regimen. Figure 5 presents the quantification of protein expression from western blot experiments in which three liver samples from individual mice (N = 3) were run to estimate a variability between biological replicates and calculate means and errors. The images presented in Supplementary Figure S1 and S2 demonstrate the respective protein levels in liver samples pooled together from three different mice per each time point for each feeding regimen and sex. For the preparation of lysates, frozen liver pieces were lysed in cell signaling lysis buffer with Protease/Phosphatase Inhibitor Cocktail (Cell Signaling Technology, Beverly, MA, USA) using sonicator. Protein concentration was determined by Bradford protein assay kit according to manufacturer’s protocol using spectrophotometer and lysates were stored at −80C. 45ug of protein was loaded on 3–8% tris-acetate and 4–12% bis-tris gels (Invitrogen). Protein was transferred on PVDF membrane at 110 mAmp. Equal loading of proteins was checked by Ponceau stain. Primary antibodies: rabbit-raised polyclonal anti-CRY1 (Signalway Antibody, 21414); rabbit-raised polyclonal anti-CRY2 (Santa Cruz Biotechnology, sc-130731); rabbit-raised monoclonal anti-FMO3 (Abcam, ab126711), mouse-raised monoclonal anti-β-ACTIN (Sigma Aldrich, A5441) were used for Immunoblot analyses. All of the primary antibodies were diluted in 5% BSA in proportions indicated in protocols by the respective manufacturers. Secondary antibodies used: Anti-rabbit IgG, HRP linked (Cell Signalling, 7074 S) and Anti-mouse IgG, HRP linked (Cell Signalling, 7076 S). Blot images were made using Scientific Imaging film and Odyssey FC imaging system (LI-COR). Protein analysis and quantification was done using Image Studio Lite Version 5.2 software.

### Statistical analysis

For all experiments, at least three male or female mice for every time point and for each feeding type were used (N = 3). Data are shown as average +/− standard error of mean. IBM SPSS Statistics 20 and GraphPad Prism Version 5.04 software packages were used for statistical analysis. To assay the effects of sex, diet and the time of the day, the analysis was performed using two-way repeated ANOVA. If the effect of feeding, time or sex was found to be statistically significant Bonferroni correction was used to calculate p-value for pairwise comparison. P < 0.05 was considered as a statistically significant difference. For the analysis of Circadian Rhythms in gene expression “R” Version 3.2.5 software – Cosinor Analysis package was used and the results are summarized in the Supplementary Tables S2 and S3.

## Electronic supplementary material

Supplementary materials
