# Supplementary material for: Calorie restriction effects on circadian rhythms in gene expression are sex dependent
Source: Sci Rep. 2017 Aug 29;7:9716. doi: 10.1038/s41598-017-09289-9 (PMC5575277; doi:10.1038/s41598-017-09289-9)
Supplement: Supplementary file 1 — Supplementary materials [file 41598_2017_9289_MOESM1_ESM.pdf]

Calorie restriction effects on circadian rhythms in gene expression are sex dependent

Artem A. Astafev, Sonal A. Patel and Roman V. Kondratov*

Department of Biological, Geological, and Environmental Sciences and Center for Gene Regulation in Health and Diseases, Cleveland State University, Cleveland, OH 44115

*Corresponding author: Roman V. Kondratov, tel.:216-523-7199, r.kondratov@csuohio.edu,

Supplementary material.

-Supplementary Table S1. Primer sequences for clock and CR-regulated/candidate longevity genes for qPCR

-Supplementary Table S2. Cosinor Wave Analysis of Clock and Longevity – Associated Gene Expression for Figures 1, 2, 3, 4 and 6.

-Supplementary Table S3. Cosinor Wave Analysis of CRY1, CRY2 and FMO3 protein expression for Figure 5.

-Supplementary Fig. S1. Sexual dimorphism in the expression of CRY1, CRY2 and FMO3 proteins

-Supplementary Fig. S2. Sex and diet effect on CRY1, CRY2 and FMO3 protein expression.

Supplementary Table S1.

Primers	Forward	Reverse
Bmal1	5'- CAC TGT CCC AGG CAT TCC A- 3'	5'- TTC CTC CGC GAT CAT TCG- 3'
Per1	5'- AGG TGG CTT TCG TGT TGG-3'	5'- CAA TCG ATG GAT CTG CTC TGA G-3'
Per2	5'- AAT CTT CCA ACA CTC ACC CC- 3'	5'- CCT TCA GGG TCC TTA TCA GTTC-3'
Per3	5'- GGT CGA CAT AAA GTC CGA ACG A- 3'	5'- TCG TTA CTG GCT GCC TTT TTT ATT- 3'
Cry1	5'- CGT CTG TTT GTG ATT CGG GG- 3'	5'- ATT CAC GCC ACA GGA GTT GC- 3'
Cry2	5'- GGC AGA CCG AGA CCC AGT CCA- 3'	5'- ATC GAT TGC GCG GGG ACC G- 3'
Rev Erb α	5'- TGG CAT GGT GCT ACT GTG TAA GG- 3'	5'- ATA TTC TGT TGG ATG CTC CGG CG- 3'
Ror γ	5'- ACT ACG GGG TTA TCA CCT GTG AG- 3'	5'- GTG CAG GAG TAG GCC ACA TTA C- 3'
Fmo3	5' CACCACCATCCAGACAGATTAC 3'	5' CCTTGAGAAACAGCCATAGGAG 3'
Cyp4a14a	5' ACGAGCACACAGATGGAGTG 3'	5' TCTTCTTCCTGGCCTTCTGC 3'
Cyp4a12b	5' CTGATGGACGTTCTTTAC 3'	5' TCAAACACCTCTGGATT 3'
Mup4	5' ACCAAAACCAATCGCTGCCT 3'	5' GCTGTATCGATCGGAAGAGAGG 3'
Serpina12	5' ACCGTGATGATTCTCACAAA 3'	5' AACATCATGGGTACCTTCAC 3'

**Igf-1**	**5’ CTGAGCTGGTGGATGCTCTT 3’**	**5’ CACTCATCCACAATGCCTGT 3’**
**18S rRNA**	**5’- GCT TAA TTT GAC TCA ACA CGG GA- 3’**	**5’- AGC TAT CAA TCT GTC AAT CCT GTC- 3’**

Supplementary Table S2.

Cosinor Wave Analysis of Clock and Longevity – Associated Gene Expression for Figures 1; 2; 3; 4 and 6. The circadian parameters were assessed by the cosinor analysis that models the circadian rhythms (Circa) as a cosine function with the following attributes: amplitude (amp), and acrophase (acr). The rhythm in gene expression pattern is considered circadian if rhythmicity fit value is greater or equal to 0.7.

****Bold italic values marked with asterisk** indicate arrhythmic expression of the respective gene for particular experimental group.

Name of Gene	**Rhythmicity fit**				**Acrophase**				**Amplitude**			
	**Male**		**Female**		**Male**		**Female**		**Male**		**Female**	
	AL	CR	AL	CR	AL	CR	AL	CR	AL	CR	AL	CR
Bmal1	0.99	0.99	0.95	0.97	22.64	22.8	22.12	22.74	19.81	23.77	71.92	27.98
Per1	0.89	0.81	0.85	0.92	12.12	11.22	12.77	11.35	11.01	8.26	10.57	18.7
Per2	0.98	0.95	0.99	0.94	14.36	15.51	14.21	14.51	8.85	15.46	13.76	19.41
Per3	0.9	0.82	0.84	0.71	12.07	10.26	10.79	10.68	11.8	13.86	29.85	27.99
Cry1	0.99	0.94	0.97	0.98	18.71	19.7	20.5	20.36	5.38	6.23	6.52	6.46
Cry2	0.94	***0.43****	***0.65****	0.72	13.51	13.86	10.98	11.17	3.76	1.92	2.19	4.29
RevErb α	0.97	0.94	0.75	0.94	7.58	7	6.8	5.78	41.12	40.99	74.98	173.91
Ror γ	0.99	0.98	0.89	0.96	16.88	18.72	18.46	18.76	4.21	4.52	6.88	5.17
Fmo3	0.84	0.98	0.84	0.85	3.91	15.69	19.47	17.43	3.23	3.24	3.29	2.16
Mup4	0.92	0.79	0.89	0.77	5.51	6.26	8.89	5.78	2.97	2.6	3.96	7.03

Serpina12	0.75	***0.66****	***0.58****	***0.57****	9.16	2.64	18.9	9.72	2.49	5.93	2.84	3.73
Cyp4a14a	***0.36****	0.72	***0.56****	***0.64****	13.56	21.66	6.59	15.42	11.78	2.14	7.2	3.81
Cyp4a12b	0.91	***0.67****	***0.53****	0.7	21.3	19.77	16.96	17.44	1.8	1.76	2.37	4.52
Igf-1	0.85	0.87	***0.65****	***0.55****	16.92	18.98	18.96	8.98	2.16	1.42	2.44	1.45

Supplementary Table S3.

Cosinor Wave Analysis of CRY1, CRY2 and FMO3 protein expression for Figure 5. The circadian parameters were assessed by the cosinor analysis that models the circadian rhythms (Circa) as a cosine function with the following attributes: amplitude (amp), and acrophase (acr). The rhythm in gene expression pattern is considered circadian if rhythmicity fit value is greater or equal to 0.7.

***Bold italic values marked with asterisk** indicate arrhythmic expression of the respective gene for particular experimental group.

**Name of Protein**	**Rhythmicity fit**				**Acrophase**				**Amplitude**			
	**Male**		**Female**		**Male**		**Female**		**Male**		**Female**	
	**AL**	**CR**	**AL**	**CR**	**AL**	**CR**	**AL**	**CR**	**AL**	**CR**	**AL**	**CR**
**CRY1**	***0.61****	0.89	***0.69****	0.93	0.96	22.70	18.98	23.03	1.87	8.36	5.32	4.07
**CRY2**	***0.42****	***0.54****	0.78	0.83	12.21	14.53	10.05	7.18	1.50	2.10	1.27	1.55
**FMO3**	***0.37****	0.72	***0.53****	0.72	12.91	12.98	23.68	15.26	1.29	1.65	1.19	1.16

Supplementary Figure S1.

Sexual dimorphism in the expression of CRY1, CRY2 and FMO3 proteins

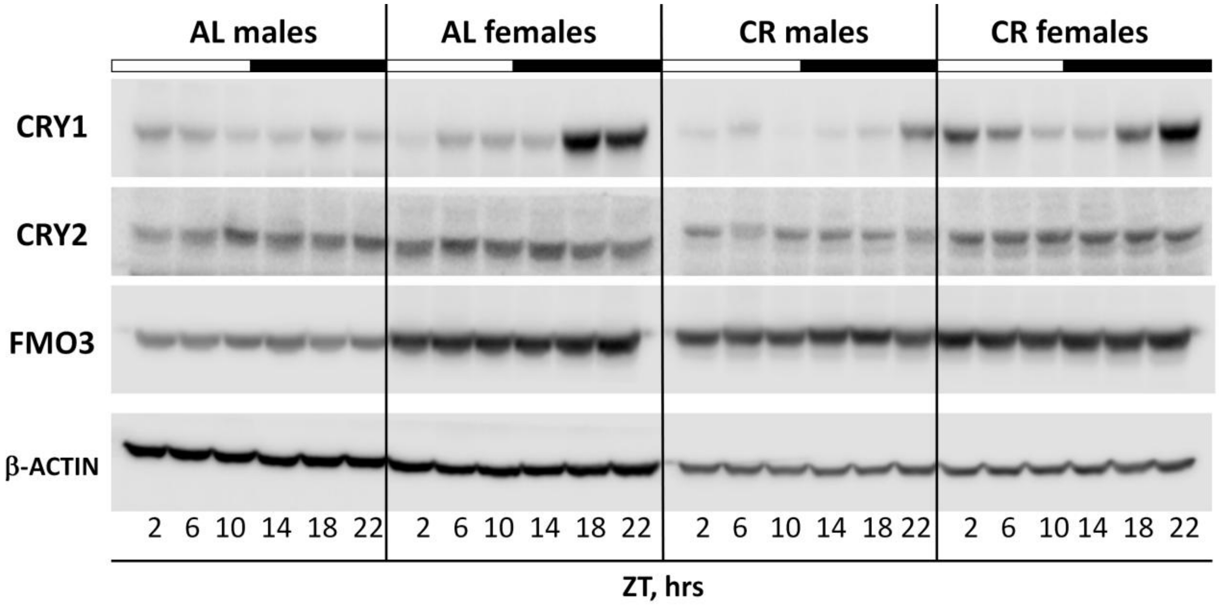

Representative Western blots for CRY1, CRY2 and FMO3 proteins assayed in the livers of mice. Liver samples from three mice were pooled together per each time point, each sex and each feeding regimen (AL - Ad libitum and CR - Caloric Restriction). Open bars and dark bars on the top of the figure represent light and dark phase of the day. The numbers on the bottom of the figure are the time points. ZT0 is the time when light is on and ZT12 is the time when light is off. Food for CR group was provided at ZT14. Lysates (loading mix) prepared from liver samples of male and female mice of the same feeding regimen throughout 6 time points are loaded on one gel for between – sex comparison. For each protein 2 blots are shown – with AL male and female samples, and with CR male and female samples.

Supplementary Figure S2.

Sex and diet effect on CRY1, CRY2 and FMO3 protein expression.

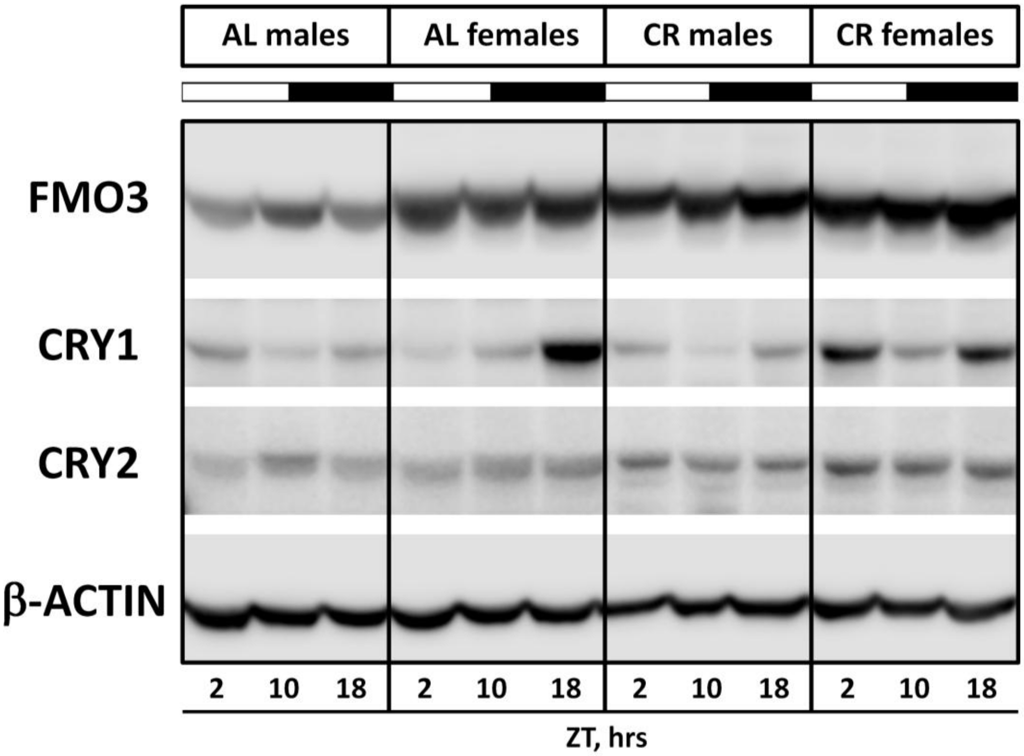

Representative Western blots for CRY1, CRY2 and FMO3 proteins assayed in the livers of mice. Liver samples from three mice were pooled together per each time point, each sex and each feeding regimen (AL - Ad libitum and CR - Caloric Restriction). Open bars and dark bars on the top of the figure represent light and dark phase of the day. The numbers on the bottom of the figure are the time points. ZT0 is the time when light is on and ZT12 is the time when light is off. Food for CR group was provided at ZT14. 3 time points out of 6 are shown, lysates (loading mix) prepared from mouse liver samples from all groups – both sexes and feeding regimen from 3 time points instead of 6 were used in order to be loaded on one gel for the demonstration of combined effect of diet

and sex on protein expression. For each protein one blot is shown with four groups of comparison loaded back – to – back.
